# Association of Surgical Approach with Intraoperative Femoral Fracture Patterns During Cementless Hemiarthroplasty for Femoral Neck Fracture

**DOI:** 10.3390/jcm15197666

**Published:** 2026-10-03

**Authors:** Ali Tecirli, Seyyid Serif Unsal, Tugrul Yildirim, Alp Karacoban

**Affiliations:** 1 Department of Orthopedics and Traumatology, Ankara Dışkapı Training and Research Hospital, Ankara 06110, Türkiye; 2 Department of Orthopedics and Traumatology, Ankara Atatürk Sanatorium Training and Research Hospital, Ankara 06290, Türkiye; seyyid.unsal@hotmail.com; 3 Department of Orthopedics and Traumatology, Yuksek Ihtisas University, Ankara 06530, Türkiye; tugrul.yildirim6@gmail.com; 4 Department of Orthopedics and Traumatology, Mardin Training and Research Hospital, Mardin 47100, Türkiye; alpkaracoban@gmail.com

**Keywords:** hemiarthroplasty, femoral neck fracture, intraoperative femoral fracture, geriatric fracture, surgical approach, greater trochanteric fracture, proximal femoral morphology

## Abstract

**Background/Objective**: Intraoperative femoral fractures during cementless hemiarthroplasty may present with distinct anatomical patterns. We examined whether surgical approach was associated with greater trochanteric versus stem-stable periprosthetic fracture patterns in older patients with osteoporotic-type femoral morphology; stem geometry was evaluated as an additional implant-related factor. **Methods**: We retrospectively reviewed 292 patients aged ≥75 years who underwent cementless bipolar hemiarthroplasty for low-energy displaced femoral neck fractures at a tertiary referral trauma center between January 2019 and July 2023. Patients were grouped according to stem geometry (fit-and-fill or tapered-wedge) and surgical approach (lateral or posterior). Associations were assessed using multivariable logistic regression, Firth-penalized regression, and stabilized inverse probability of treatment weighting (IPTW). **Results**: Intraoperative fractures occurred more frequently with the lateral approach than with the posterior approach (23.7% vs. 12.2%; *p* = 0.015), primarily because of greater trochanteric fractures (9.5% vs. 2.4%; *p* = 0.016). Periprosthetic fracture rates did not differ significantly between the approaches (14.2% vs. 9.8%; *p* = 0.284). In the Firth model, the posterior approach was associated with lower odds of greater trochanteric fracture (OR 0.25, 95% CI 0.08–0.80; *p* = 0.019); after IPTW, the corresponding estimate was OR 0.19 (95% CI 0.05–0.76; *p* = 0.019). **Conclusions**: The surgical approach was primarily associated with greater trochanteric fractures, whereas no independent association was observed with stem geometry. Pattern-specific analysis may provide a clearer characterization of intraoperative fracture associations in this setting.

## 1. Introduction

Hemiarthroplasty is a standard treatment for displaced femoral neck fractures in older adults and is supported by current clinical guidelines [1]. In this setting, intraoperative mechanical complications are especially relevant because osteoporotic bone may be vulnerable to both the forces generated during exposure and press-fit femoral preparations.

Cementless femoral fixation is widely used in hip arthroplasty and has also been studied extensively in older and fracture populations [2,3], but press-fit implantation introduces a risk of femoral fracture [4,5]. In older patients with osteoporotic-type femoral morphology, the extent to which this risk is related to implant geometry and surgical exposure remains uncertain [6].

Previous studies have emphasized both implant-related and approach-related contributors. Registry studies and large clinical series have reported more periprosthetic fractures with cementless fixation, particularly in older patients and those with unfavorable femoral morphology [7,8,9,10]. Advanced age has also been associated with intraoperative periprosthetic fractures during cementless hemiarthroplasty [11]. Other studies have emphasized the femoral component design and found less influence from the surgical approach [12,13,14]. In contrast, approach-focused studies suggest that exposure, femoral elevation, and manipulation can affect the location and mechanism of fracture [15,16,17,18]. Intraoperative periprosthetic fractures have also been described in dedicated hemiarthroplasty cohorts, although the determinants of individual fracture patterns are still not well defined [19].

These studies address overlapping questions but do not fully resolve the issues. Parker’s randomized trial directly compared lateral and posterior hemiarthroplasty approaches and found broadly comparable functional outcomes, while the lateral approach was judged technically easier [15]. Sershon et al., in a population closer to elective arthroplasty practice, reported a stronger effect of stem choice than of approach on periprosthetic fracture risk [12]. A secondary analysis of the Hip Fracture Evaluation with Alternatives of Total Hip Arthroplasty versus Hemi-Arthroplasty (HEALTH) trial evaluated stem design and approach in patients with femoral neck fractures; however, it did not separate intraoperative fractures according to anatomical pattern [6]. Recently, Rust et al. examined fixation and approach in a large single-center hemiarthroplasty cohort; their outcomes focused on postoperative complications, recovery, discharge, and mortality rather than on pattern-specific intraoperative femoral fractures [20].

This distinction may be important in osteoporotic femurs. Greater trochanteric fractures may plausibly be influenced by exposure-related forces and femoral manipulation [16,17,18], whereas stem-adjacent fractures may be more dependent on canal shape, implant fit, and canal fill [3,5,21,22,23,24]. Experimental work on stem geometry and primary stability [22], together with morphology-based clinical studies [23,24], supports the notion that these injuries may not share a single mechanism. The clinical consequences of these differences may also vary [25]. Therefore, we analyzed greater trochanteric and periprosthetic femoral fractures separately rather than treating every intraoperative fracture as the same.

Our primary aim was to assess the association of surgical approach with specific intraoperative femoral fracture patterns in older adults with osteoporotic-type femoral morphology undergoing cementless hemiarthroplasty for femoral neck fracture. Stem geometry was evaluated as an additional implant-related factor rather than as the principal comparative focus. We also examined the lateral and posterior approaches in relation to the two principal fracture patterns. We expected greater trochanteric fractures to show a stronger association with surgical approach, whereas analyses involving stem geometry and the AL/B1 endpoint were considered secondary within this pattern-focused framework.

## 2. Materials and Methods

### 2.1. Study Design and Patient Selection

**Figure 1:**
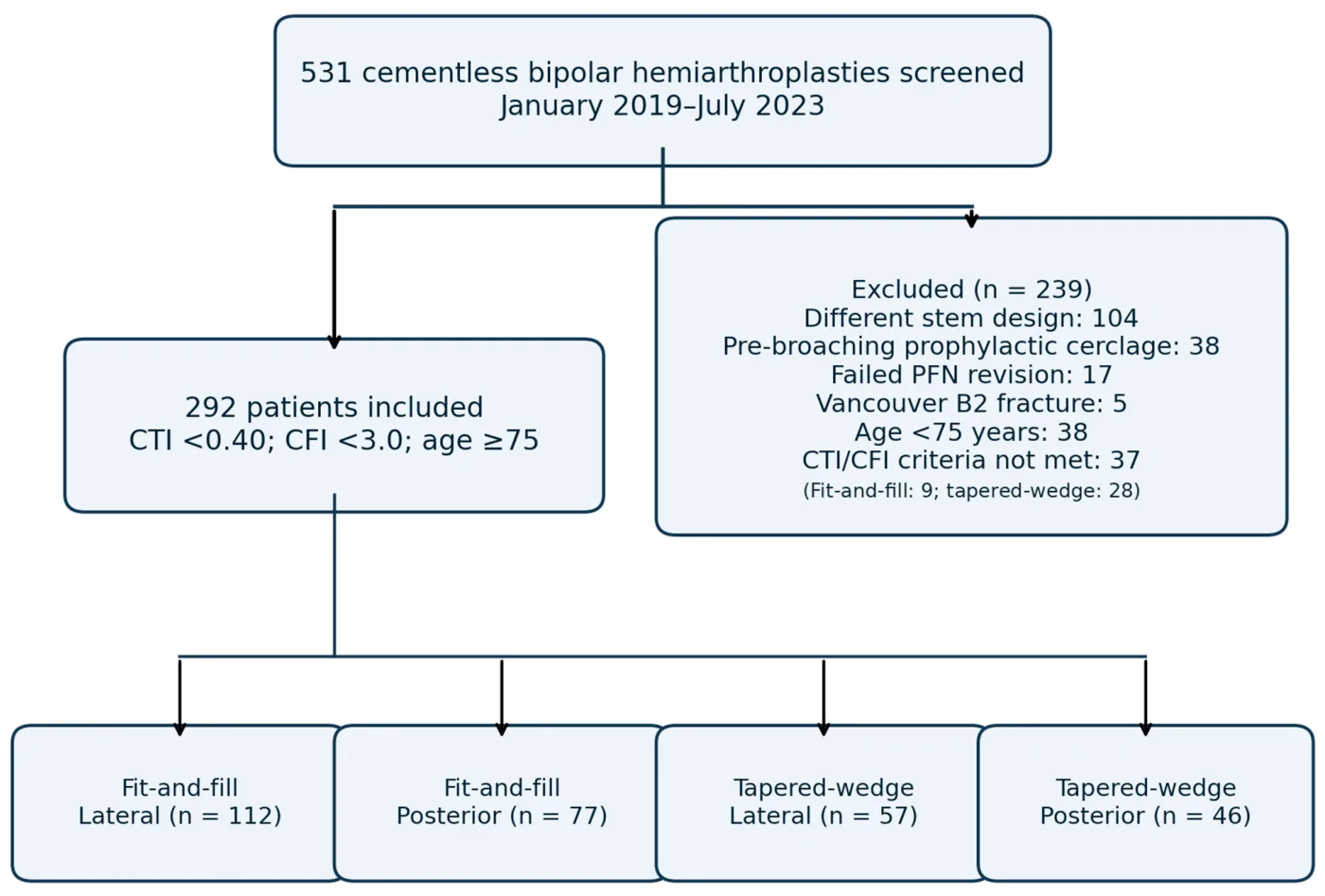
Flow diagram of patient selection and cohort formation. Of the 37 excluded for failing the CTI/CFI criteria, 9 had fit-and-fill stems and 28 had tapered-wedge stems. The final analytical cohort met age ≥ 75 years, CTI < 0.40, and CFI < 3.0 criteria. CTI, cortical thickness index; CFI, canal flare index; PFN, proximal femoral nail.

This single-center retrospective cohort study included patients treated at Dışkapı Training and Research Hospital, a tertiary referral trauma center, between January 2019 and July 2023. Among the 531 cementless bipolar hemiarthroplasties screened, 239 cases were excluded: 104 had a femoral stem design outside the two study groups; 38 Taperloc cases received planned prophylactic cerclage before femoral broaching; 17 underwent revision hemiarthroplasty after failed proximal femoral nail fixation; 5 had Vancouver type B2 fractures; 38 patients were younger than 75 years; and 37 did not meet the prespecified radiographic morphology criteria (CTI < 0.40 and CFI < 3.0; fit-and-fill, *n* = 9; tapered-wedge, *n* = 28). The 38 prophylactically cabled cases were excluded on surgical-technique grounds before radiographic morphology screening; CTI and CFI eligibility was not assessed in this group. The final cohort comprised 292 patients (Figure 1). Complete baseline clinical and radiographic data were available for all variables used in primary models. The CTI and CFI thresholds were used to identify an osteoporotic-type proximal femoral morphology. They were radiographic eligibility criteria rather than a formal diagnosis of osteoporosis or a formal Dorr classification. Their use was informed by the classic proximal femoral morphology framework described by Dorr et al. [26] and contemporary morphology-based literature [24]. Institutional permission to access and use the retrospective clinical data was obtained from Dışkapı Training and Research Hospital on 8 May 2026, followed by ethical approval from the Liv Hospital Ankara Ethics Committee on 12 May 2026 (approval no. 2026/034). Data extraction, radiographic measurements, and statistical analyses were initiated only after these approvals had been obtained.

### 2.2. Exclusion Criteria

Planned prophylactic cerclage placed before femoral broaching was an exclusion criterion applied before radiographic morphology screening. Because the cable alters the mechanical conditions of femoral preparation, including these cases in the analysis of intraoperative fracture patterns by surgical approach and stem geometry could confound the associations of interest. All 38 such cases received a Taperloc stem; 23 had a lateral and 15 had a posterior approach. The remaining exclusions were for a different stem design, failed proximal femoral nail revision, Vancouver type B2 fracture, age < 75 years, or failure to meet CTI < 0.40 and CFI < 3.0. Exclusion categories in Figure 1 are reported as separate counts. No additional exclusions were made for missing analytical data.

### 2.3. Surgeons and Selection Bias

All procedures were performed by orthopedic surgeons working at the same study institution. Both the lateral and posterior approaches were routinely used at the institution, and the surgeons were familiar with both techniques. The choice of approach was not determined by a study protocol or randomized allocation; rather, it reflected the operating surgeon’s routine practice and preference. Surgeon identifiers, surgeon-specific case volumes, and individual surgeon experience were not recorded in the analytical dataset. Therefore, we could not determine how frequently individual surgeons used each approach, whether some surgeons predominantly favored one approach, or directly adjust for surgeon-level clustering. Surgeon-related factors were consequently treated as a potential source of residual confounding.

### 2.4. Implant Characteristics

All femoral components were manufactured by Biomet (Warsaw, IN, USA) and Stryker (Stryker Corporation, Kalamazoo, MI, USA). For the analysis, the implants were grouped by geometry rather than being treated as a commercial comparison. One family had a fit-and-fill design intended to increase metaphyseal and canal conformity, and the other had a tapered wedge design relying mainly on metaphyseal press-fit fixation. Because stem geometry can alter primary stability and load transfer [22], and because clinical performance may depend on the interaction between the implant and femoral morphology [24], we focused on these design principles rather than brand-level differences in our study.

### 2.5. Standardization of Surgical Technique

Femoral preparation followed the same general institutional principles for both approaches: a controlled entry point, sequential broaching until press-fit stability was achieved, avoidance of varus alignment, and limitation of excessive torsional loading during femoral elevation. With the lateral approach, particular care was taken to preserve the abductor complex and the trochanteric attachments. With the posterior approach, preparation was performed with controlled external rotation and elevation. Operative records were reviewed for major deviations, but several technical details could not be consistently reconstructed from the retrospective records, including exact broach increments, reaming, use of elevation aids, retractor position, and magnitude of manipulation. Stem alignment was measured on the first postoperative anteroposterior radiograph as the coronal angle between the stem axis and the anatomical femoral axis. Varus malposition was defined a priori as coronal varus ≥ 3°.

### 2.6. Radiographic Measurement Reliability

Interobserver reliability was examined in 150 patients with complete paired radiographic measurements obtained independently by two observers. The analysis covered the CFI, canal–calcar ratio (CCR), proximal and distal canal fill ratios, CTI, and canal–bone ratio. Agreement was quantified using the intraclass correlation coefficient [ICC(2,1)], corresponding to a two-way random-effects, absolute-agreement, single-measure model, with 95% confidence intervals.

### 2.7. Fracture Definition and Classification Approach

Only fractures recognized intraoperatively and documented in the operative record were included as intraoperative femoral fractures. Immediate postoperative radiographs obtained during the index admission were reviewed together with the operative notes to confirm the fracture pattern and implant position; no additional fracture that had not been recognized intraoperatively was identified on these radiographs. Fracture ascertainment and Vancouver classification were performed jointly by two orthopedic surgeons (A.T. and S.S.U.) through review of the operative notes and radiographs, and classification decisions were reached by consensus. The reviewers were not blinded to stem geometry, which was visible on the radiographs, or to surgical approach, which was obtained from the operative record. Fractures were classified as AG (greater trochanter), AL (lesser trochanter/calcar-adjacent), or B1 (stem-stable) [27,28,29]. When more than one anatomical fracture component was present in the same patient, the case was assigned once to the dominant fracture pattern judged to represent the principal intraoperative injury requiring management; therefore, the pattern-specific categories were mutually exclusive in the analytical dataset. AG fractures were analyzed as greater trochanteric fractures. Calcar-adjacent fractures with preserved stem stability were classified as AL; cerclage fixation was used intraoperatively when required to maintain stability. Stem stability for B1 classification was determined from the operative documentation together with the postoperative clinical and weight-bearing records. AL and B1 fractures were combined as a stem-stable periprosthetic endpoint for the primary pattern-specific analysis because they shared a stable-stem clinical context and the same immediate management in this cohort, while their individual raw counts were also reported separately for transparency. In all 34 AL fractures and both B1 fractures, femoral stem stability was preserved, and the fractures were managed with cerclage cable fixation without femoral stem revision.

### 2.8. Statistical Analysis

Data distributions were assessed within each of the four stem-by-approach groups using the Shapiro–Wilk test. Continuous variables were summarized as mean ± standard deviation when the distribution was compatible with normality across all four groups and as median (minimum–maximum) otherwise; categorical variables were summarized as counts and percentages. Baseline characteristics across the four stem-by-approach groups were compared using one-way analysis of variance (ANOVA) for normally distributed continuous variables and the Kruskal–Wallis test for non-normally distributed continuous variables. When a global group comparison was statistically significant, Tukey’s honestly significant difference test was used for post hoc pairwise comparisons after ANOVA, whereas Dunn’s pairwise test with Bonferroni correction was used after the Kruskal–Wallis test. Categorical variables were compared using the chi-square test or Fisher’s exact test, as appropriate. No a priori sample-size calculation was performed because this retrospective study included all eligible patients treated during the predefined period; consequently, the precision and statistical power of pattern-specific analyses were constrained by the observed number of fracture events.

For the primary approach analysis, the two stem families were pooled and the lateral and posterior approaches were compared directly. The primary multivariable logistic regression model for any intraoperative femoral fracture included surgical approach (posterior vs. lateral), stem geometry (tapered-wedge vs. fit-and-fill), age (continuous), sex (female vs. male), age-adjusted Charlson Comorbidity Index (aCCI; continuous), and canal flare index (CFI; continuous). Surgical approach was the primary exposure of interest; stem geometry was retained as an additional implant-related factor, while age, sex, and aCCI were retained as clinically plausible confounders. CFI was selected as the principal continuous femoral morphology covariate because the radiographic morphology indices were correlated and CTI was already part of the eligibility definition, thereby limiting collinearity and overparameterization. A stem-by-approach interaction term was tested for possible effect modification. Varus malposition, defined a priori as coronal stem varus ≥ 3° on the immediate postoperative anteroposterior radiograph, was not included in the primary adjustment set because it was a postoperative stem-position variable that could represent an intermediate or consequence of the operative process. To assess the robustness of this assumption, a prespecified revision sensitivity model added binary varus malposition (≥3° vs. <3°) to the primary logistic regression model.

For lower-frequency fracture outcomes, specifically Vancouver AG and the combined AL/B1 endpoint, Firth bias-reduced logistic regression was used to reduce small-sample bias and the risk of separation. The Firth models used the same core covariate structure as the conventional outcome model. Estimation was implemented in Python using the Jeffreys-prior adjusted-score algorithm; 95% confidence intervals and two-sided *p* values were calculated using Wald inference based on the inverse penalized information matrix.

Inverse probability of treatment weighting (IPTW) was used as a robustness analysis for the posterior-versus-lateral approach comparison. Propensity scores for receipt of the posterior approach were estimated with binary logistic regression including age, sex, aCCI, American Society of Anesthesiologists (ASA) Physical Status Classification (≥3 vs. <3), stem geometry, and CFI. Stabilized average-treatment-effect (ATE) weights were calculated and raw weights were truncated at the 1st and 99th percentiles. Covariate balance before and after weighting was evaluated using standardized mean differences (SMDs), with |SMD| < 0.10 considered adequate balance. IPTW outcome estimates were obtained from weighted binomial generalized linear models with HC0 robust (sandwich) covariance for variance estimation. An exploratory multinomial weighting analysis across the four stem-by-approach groups was also attempted, but adequate overlap and balance could not be achieved across all radiographic covariates; therefore, this analysis was not used for primary inference regarding stem effects. Surgeon-level mixed models or cluster-robust standard errors could not be fitted because surgeon identifiers were unavailable.

Analyses for the revision were performed using Python 3.13.5 with NumPy 2.3.5, SciPy 1.17.0, Statsmodels 0.14.6, and Scikit-learn 1.8.0. All tests were two-sided, with *p* < 0.05 considered statistically significant. Secondary and exploratory analyses were not formally adjusted for multiple comparisons except for the prespecified Bonferroni correction used in Dunn post hoc comparisons and should therefore be interpreted cautiously. The completed STROBE checklist for this retrospective cohort study is provided in the Supplementary Materials.

## 3. Results

Table 1 summarizes the baseline clinical characteristics, radiographic morphology, and major intraoperative events across the four stem-by-approach groups. Before adjustment, significant between-group differences were present in CFI, CCR, proximal and distal canal fill ratios, CTI, and CBR, whereas age, aCCI, sex, ASA category, and varus malposition did not differ significantly across the four groups. These global four-group comparisons are distinct from the pooled lateral-versus-posterior outcome comparisons reported below.

The four-group *p* values presented in Table 1 represent global comparisons across the four approach-stem combinations, whereas the pooled lateral-versus-posterior analyses collapse across stem geometry and therefore evaluate a different contrast.

The agreement between the observers was excellent for all six radiographic measurements in the 150-patient reliability subset. Using the ICC(2,1) model defined above, the coefficients were 0.984 for the CFI, 0.978 for the CCR, 0.971 for the proximal canal fill ratio, 0.926 for the distal canal fill ratio, 0.974 for the CTI, and 0.995 for the canal–bone ratio (all *p* < 0.001).

When the stem families were pooled by approach, intraoperative femoral fractures occurred in 23.7% and 12.2% of lateral and posterior cases, respectively (*p* = 0.015). The difference was more pronounced for greater trochanteric fractures (9.5% vs. 2.4%; *p* = 0.016). Periprosthetic femoral fracture rates were numerically higher with the lateral approach but did not differ significantly between the two approaches (14.2% vs. 9.8%; *p* = 0.284). Among the 36 AL/B1 events, 34 were AL (calcar/lesser-trochanteric) fractures and 2 were stem-stable B1 fractures. The unadjusted and adjusted estimates are shown in Table 2.

In the conventional multivariable model, the posterior approach was associated with lower odds of intraoperative fracture (OR 0.40, 95% CI 0.20–0.78; *p* = 0.007). Stem design was not independently associated with the outcome (tapered-wedge vs. fit-and-fill: OR 0.87, 95% CI 0.45–1.65; *p* = 0.660). CFI showed an association with overall fracture risk (OR 3.50, 95% CI 1.03–11.87; *p* = 0.044), although the confidence interval remained wide. In the prespecified sensitivity analysis, adding varus malposition (≥3°) to the primary model left the posterior-versus-lateral estimate essentially unchanged (OR 0.40, 95% CI 0.20–0.78; *p* = 0.007; relative OR change −0.03%); varus malposition itself was not independently associated with the outcome (OR 1.04, 95% CI 0.32–3.35; *p* = 0.953).

There was no statistically significant stem-by-approach interaction (OR 0.89, 95% CI 0.22–3.59; *p* = 0.869), although the wide confidence interval limited the precision of the estimate.

For greater trochanteric fractures, the Firth-penalized model again showed lower odds with the posterior approach (OR 0.25, 95% CI 0.08–0.80; *p* = 0.019), while stem design was not independently associated with this endpoint (OR 1.51, 95% CI 0.61–3.75; *p* = 0.375). For AL/B1 periprosthetic fractures, the association with the posterior approach was smaller and not statistically significant (OR 0.60, 95% CI 0.29–1.26; *p* = 0.179). Stem geometry was likewise not significantly associated with the AL/B1 endpoint (OR 0.65, 95% CI 0.30–1.41; *p* = 0.273), and CFI remained imprecisely estimated (OR 3.70, 95% CI 0.88–15.51; *p* = 0.073).

**Figure 2:**
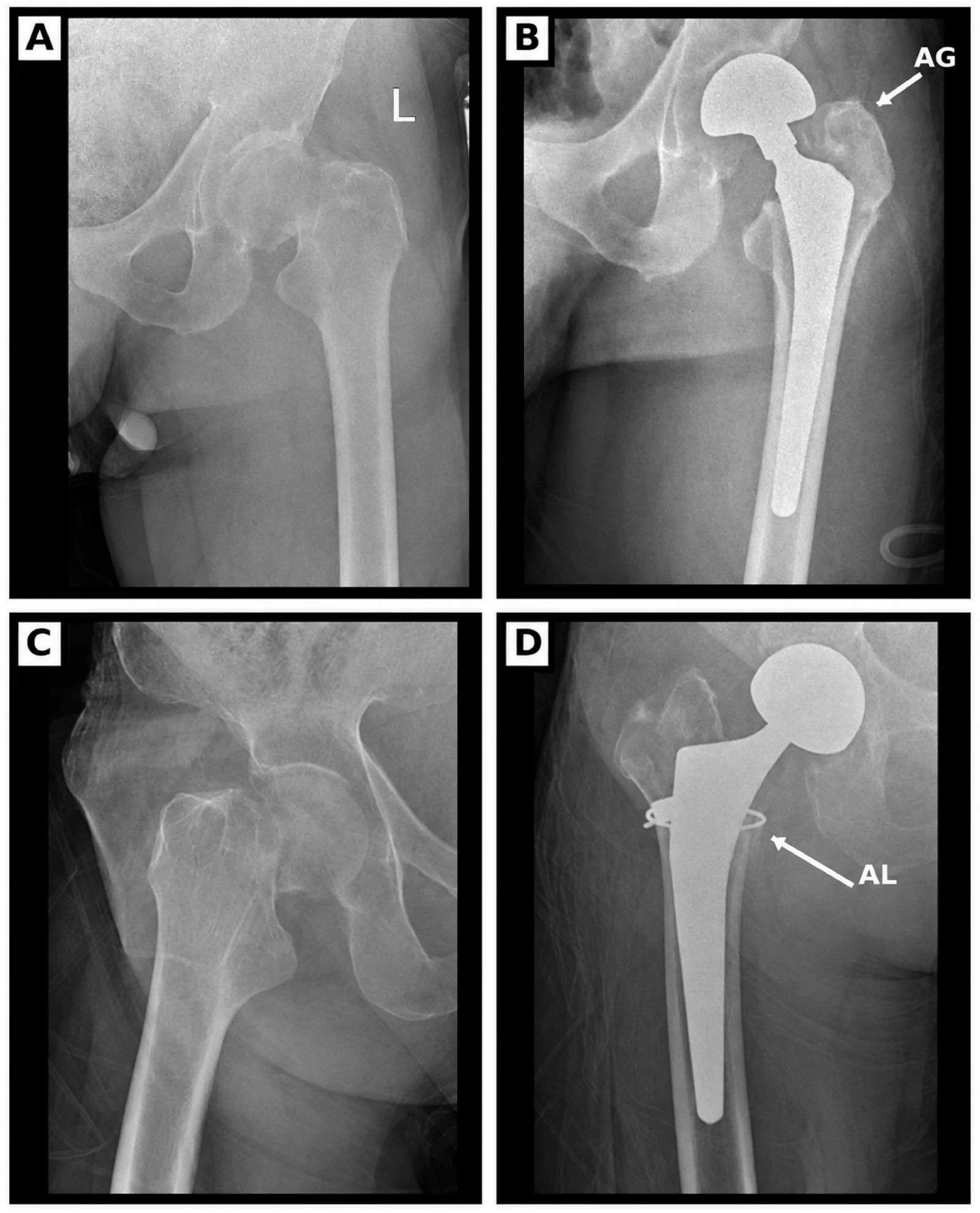
Representative radiographs of intraoperative femoral fracture patterns after cementless hemiarthroplasty. (**A**) Preoperative anteroposterior radiograph of a patient with a displaced femoral neck fracture. (**B**) Immediate postoperative radiograph demonstrating an isolated Vancouver AG greater trochanteric fracture (arrow). (**C**) Preoperative anteroposterior radiograph of a second patient with a displaced femoral neck fracture. (**D**) Immediate postoperative radiograph demonstrating a Vancouver AL calcar/lesser-trochanteric fracture extending adjacent to the proximal stem (arrow), stabilized with a cerclage cable. L indicates the left side.

After inverse probability of treatment weighting (IPTW), all measured covariates met the prespecified balance criterion (all post-weighting |SMD| < 0.10). The posterior approach was associated with lower odds of any intraoperative fracture (OR 0.41, 95% CI 0.21–0.79; *p* = 0.008) and greater trochanteric fracture (OR 0.19, 95% CI 0.05–0.76; *p* = 0.019). No statistically significant association was observed between surgical approach and AL/B1 periprosthetic fracture after weighting (OR 0.61, 95% CI 0.29–1.29; *p* = 0.197). Table 2 summarizes the unadjusted, multivariable, Firth, and IPTW estimates. The exploratory four-group propensity analysis did not achieve the prespecified balance threshold for all radiographic covariates; therefore, it was not used for primary inference regarding stem effects. Representative radiographs illustrating the fracture patterns are shown in Figure 2.

## 4. Discussion

In this study, the term osteoporotic-type femoral morphology refers to the prespecified radiographic criteria (CTI < 0.40 and CFI < 3.0) and should not be interpreted as a formal diagnosis of osteoporosis. Bone mineral density measurements and T-scores were not available for all patients.

The main finding was not simply a difference in the overall fracture rate but also a difference in fracture pattern. Among older patients selected for osteoporotic-type femoral morphology, greater trochanteric fractures were more frequent with the lateral approach, and this association remained after multivariable adjustment and IPTW. The posterior approach was associated with lower odds of these fractures, but this finding represents a relative association rather than elimination of fracture risk. We did not identify an independent association between stem geometry and either the overall fracture endpoint or the individual fracture patterns. The AL/B1 endpoint behaved differently: neither approach nor stem design reached statistical significance, and morphology-related estimates were suggestive but imprecise.

The present study was designed primarily as a fracture-pattern and surgical-approach analysis rather than as a comparative evaluation of femoral stem performance. Stem geometry was examined as an additional implant-related factor within this framework.

The four groups differed in several radiographic measures at baseline, including CFI, CTI, and canal fill. This is important because femoral morphology can influence both implant selection and fracture susceptibility. In the pooled approach comparison, IPTW achieved the prespecified balance target, and the association between approach and greater trochanteric fracture persisted after weighting. This finding reduces, but does not eliminate, concern that the observed association was explained by the measured covariates. Residual confounding remains possible, particularly from surgeon preference, exposure technique, implant selection, and other technical factors that could not be reconstructed or modeled at the operator level.

Our results sit between two lines of literature that are often discussed separately: registry studies and large clinical series emphasize the periprosthetic fracture burden of cementless fixation [7,8,9,10], whereas studies on surgical approaches emphasize exposure and femoral manipulation [15,16,17,18]. The present data suggest that both may be relevant, but to different fracture patterns. Greater trochanteric injuries may be more sensitive to exposure-related forces, whereas stem-adjacent injuries may depend more on femoral morphology and implant fit. Because the consequences of these fractures also differ [25], combining them into one endpoint can hide clinically useful information.

One plausible explanation for the observed association is that differences in femoral exposure, elevation, retractor positioning, broaching, or manipulation may alter loading of the greater trochanter. However, these technical factors were not quantitatively assessed in the present retrospective study. Approach-specific perioperative fracture risk has been examined in cementless THA cohorts using direct anterior or anterolateral exposures [16,17,18], but these studies do not reproduce our lateral-versus-posterior hemiarthroplasty comparison. Accordingly, the proposed biomechanical explanation should be regarded as hypothesis-generating rather than as a mechanism directly demonstrated by our data.

This finding should not be interpreted as an argument against the lateral approach to the hip joint, nor should the lower fracture odds observed with the posterior approach be interpreted as absence of intraoperative fracture risk. The observed association suggests that careful femoral exposure and controlled manipulation may be particularly relevant when the lateral approach is used in older patients with osteoporotic-type femoral morphology; however, this technical interpretation is based on a plausible biomechanical hypothesis rather than on a mechanism directly demonstrated in the present study. Fractures recognized intraoperatively in our cohort were stabilized with cables when clinically indicated. Because patients with prophylactic cerclage cable use were excluded from the analytical cohort, the present study cannot evaluate whether prophylactic cabling reduces fracture risk.

The AL/B1 endpoint was less clear. Neither the surgical approach nor stem geometry was independently associated with these fractures, and the estimate for CFI was imprecise and did not reach statistical significance. Previous studies on canal shape, stem fit, Dorr type, and canal fill provide a morphology-based framework for considering this fracture pattern [3,5,21,22,23,24]. Our data are compatible with such a framework but do not identify or establish specific risk factors for AL/B1 fractures. Femoral morphology and implant fit therefore remain plausible contributors that require confirmation in larger cohorts. We combined AL and stem-stable B1 fractures because all 34 AL fractures and both B1 fractures retained stem stability and were managed with cerclage cable fixation without stem revision; however, this remains a pragmatic simplification of two anatomically distinct injuries, and the individual event counts are reported separately.

The absence of a statistically significant association with stem geometry should not be interpreted as evidence of equivalence, superiority, or inferiority of either stem design. Event numbers were limited within the fracture subtypes, and the stem-by-approach groups differed in baseline morphology. In addition, both implants were established press-fit cementless designs; therefore, the present study was not designed to support a preference for one stem geometry over the other [12,13,14].

The propensity analyses also clarified the limits of inference. In the pooled approach comparison, weighting achieved the prespecified balance target, and the association with greater trochanteric fracture persisted. In contrast, the exploratory four-group weighting analysis did not achieve adequate common support and covariate balance across all radiographic variables. Accordingly, inference regarding stem geometry is less robust than the approach comparison and rests primarily on regression adjustment rather than on an equally well-balanced propensity analysis.

We found no statistically significant stem-by-approach interaction. This does not prove that the effect of the approach is identical across stem families because the interaction estimates were imprecise. Therefore, the practical message is modest: protection of the trochanteric region remains relevant during femoral preparation in osteoporotic bone, regardless of the stem family used.

This study has several limitations. Its retrospective observational design leaves the possibility of selection bias, residual confounding, and unmeasured confounding. Prophylactic cerclage was excluded because placement before broaching changed the operative conditions relevant to intraoperative fracture. Surgeons may have selected prophylactic cabling for patients perceived to be at greater fracture risk; all 38 excluded cases received a Taperloc stem, and their approaches were distributed as 23 lateral and 15 posterior. Their exclusion may therefore create selection bias and limits generalizability to procedures without planned prophylactic cerclage. The study cannot estimate the effect of prophylactic cabling itself. Baseline femoral morphology differed across the four stem-by-approach groups, and exploratory multinomial weighting did not fully resolve these differences. Although the surgeons at the institution were familiar with both lateral and posterior approaches, the approach used in an individual case reflected the operating surgeon’s routine practice and preference. Because surgeon identifiers and surgeon-specific case volumes were unavailable, we could not determine whether individual surgeons used both approaches with similar frequency or whether some surgeons predominantly favored one approach. Residual confounding by surgeon preference and surgeon-specific exposure, femoral elevation, retractor positioning, broaching, or manipulation techniques therefore cannot be excluded. These technical factors were not quantitatively captured in the retrospective data. Event numbers were limited for the pattern-specific outcomes (AG, *n* = 19; AL/B1, *n* = 36), reducing precision and increasing the possibility of type II error. Eligibility was defined using CTI < 0.40 and CFI < 3.0 rather than formal patient-level Dorr classification. Consequently, the findings should not be directly extrapolated to younger patients with better bone quality or to femora with more robust cortical morphology, including typical Dorr A or B patterns. Finally, AL and B1 fractures were combined into a clinically pragmatic stem-stable endpoint, and secondary and exploratory analyses were not formally adjusted for multiple comparisons. Borderline findings should therefore be interpreted cautiously.

This study also has several strengths. It focuses on a relatively homogeneous older fracture population selected with prespecified radiographic criteria, separates intraoperative fractures by clinically meaningful patterns, and evaluates the primary approach comparison using both conventional multivariable adjustment and propensity weighting. This design allowed us to examine whether a pooled fracture endpoint masked the different associations across fracture types.

## 5. Conclusions

In this single-center retrospective cohort study of older patients with osteoporotic-type femoral morphology, surgical approach was associated with the pattern of intraoperative femoral fracture, particularly greater trochanteric fracture, whereas no independent association was identified with stem geometry. The lower odds observed with the posterior approach represent a relative association and should not be interpreted as absence of fracture risk. The AL/B1 endpoint showed a different and less definitive pattern; femoral morphology and implant fit remain plausible contributors, but the present models do not establish specific risk factors. The findings regarding stem geometry are less robust than those for surgical approach and do not support claims of superiority, inferiority, or equivalence between the two stem designs. Any proposed explanation involving trochanteric loading or femoral manipulation should be regarded as hypothesis-generating, because these technical factors were not measured quantitatively. Pattern-specific analysis may therefore provide more clinically informative interpretation than treating all intraoperative femoral fractures as a single homogeneous complication category. These associations should be confirmed in larger multicenter or multi-surgeon cohorts in which surgeon-level data can be incorporated explicitly.

## Figures and Tables

**Table 1:** Baseline clinical characteristics, radiographic morphology, and major intraoperative events across the four subgroups.

Variable	*p*	Fit-and-Fill/Lateral (*n* = 112)	Fit-and-Fill/Posterior (*n* = 77)	Tapered-Wedge/Lateral (*n* = 57)	Tapered-Wedge/Posterior (*n* = 46)
**Demographic and clinical variables**					
Age	0.538	80.00 [75.00–96.00]	79.00 [75.00–93.00]	80.00 [75.00–90.00]	80.00 [75.00–90.00]
aCCI	0.866	4.00 [2.00–9.00]	4.00 [2.00–9.00]	4.00 [2.00–7.00]	4.00 [2.00–6.00]
Female	0.983	68/112 (60.7%)	46/77 (59.7%)	36/57 (63.2%)	28/46 (60.9%)
Male		44/112 (39.3%)	31/77 (40.3%)	21/57 (36.8%)	18/46 (39.1%)
ASA ≥ 3	0.129	105/112 (93.8%)	68/77 (88.3%)	55/57 (96.5%)	45/46 (97.8%)
Malposition angle (°)	0.141	1.60 [0.20–5.00]	1.30 [0.00–5.70]	1.20 [0.00–4.30]	1.10 [0.00–5.10]
**Radiographic morphology**					
Varus malposition (≥3°)	0.630	8/112 (7.1%)	7/77 (9.1%)	2/57 (3.5%)	4/46 (8.7%)
CFI	0.001	2.50 [1.60–2.97]	2.57 [2.07–2.99]	2.42 [1.81–2.99]	2.60 [1.81–2.99]
CCR	0.001	0.66 ± 0.08	0.68 ± 0.08	0.65 ± 0.07	0.62 ± 0.07
CFRprox	<0.001	0.71 ± 0.07	0.70 ± 0.06	0.68 ± 0.06	0.66 ± 0.06
CFRdist	0.040	0.88 [0.71–0.97]	0.87 [0.71–0.96]	0.86 [0.67–0.95]	0.85 [0.71–0.97]
CTI	0.003	0.26 ± 0.06	0.29 ± 0.06	0.27 ± 0.05	0.27 ± 0.05
CBR	0.003	0.74 ± 0.06	0.71 ± 0.06	0.73 ± 0.05	0.73 ± 0.05
**Major intraoperative events**					
Greater trochanter fracture	0.075	9/112 (8.0%)	2/77 (2.6%)	7/57 (12.3%)	1/46 (2.2%)
AL fracture (calcar/lesser trochanter)	—	18/112 (16.1%)	8/77 (10.4%)	4/57 (7.0%)	4/46 (8.7%)
B1 fracture (stem-stable)	—	1/112 (0.9%)	0/77 (0.0%)	1/57 (1.8%)	0/46 (0.0%)
Periprosthetic femoral fracture (AL/B1)	0.293	19/112 (17.0%)	8/77 (10.4%)	5/57 (8.8%)	4/46 (8.7%)
Any intraoperative fracture	0.086	28/112 (25.0%)	10/77 (13.0%)	12/57 (21.1%)	5/46 (10.9%)

Note. Continuous variables are presented in a single format according to distribution: mean ± standard deviation for variables meeting the group-specific normality criterion and median [minimum–maximum] otherwise. Global four-group *p* values were obtained using one-way ANOVA for normally distributed continuous variables, Kruskal–Wallis tests for non-normally distributed continuous variables, and chi-square or exact tests for categorical variables, as appropriate. Significant global continuous-variable comparisons were followed by Tukey HSD after ANOVA or Dunn pairwise tests with Bonferroni correction after Kruskal–Wallis testing. Individual AL and B1 rows are descriptive raw counts and were not tested separately because of the very small B1 event count; the prespecified inferential endpoint was AL/B1 combined. Varus malposition was defined as coronal varus alignment ≥3°. CFI, canal flare index; CCR, canal–calcar ratio; CFRprox, proximal canal fill ratio; CFRdist, distal canal fill ratio; CTI, cortical thickness index; CBR, canal–bone ratio.

**Table 2:** Unadjusted rates, multivariable models, and IPTW analyses for intraoperative fracture patterns.

Model/Outcome	Unadjusted Rates	Comparison	OR	95% CI	*p*
Conventional logistic	15/123 (12.2%) vs. 40/169 (23.7%)	Any intraoperative fracture: Posterior vs. lateral	0.40	0.20–0.78	0.007
Conventional logistic	17/103 (16.5%) vs. 38/189 (20.1%)	Any intraoperative fracture: tapered-wedge vs. fit-and-fill	0.87	0.45–1.65	0.660
Interaction model	—	Stem-by-approach interaction	0.89	0.22–3.59	0.869
Sensitivity logistic	15/123 (12.2%) vs. 40/169 (23.7%)	Any intraoperative fracture: Posterior vs. lateral, additionally adjusted for varus ≥ 3°	0.40	0.20–0.78	0.007
Firth logistic	3/123 (2.4%) vs. 16/169 (9.5%)	Greater trochanter fracture: Posterior vs. lateral	0.25	0.08–0.80	0.019
Firth logistic	8/103 (7.8%) vs. 11/189 (5.8%)	Greater trochanter fracture: tapered-wedge vs. fit-and-fill	1.51	0.61–3.75	0.375
Firth logistic	12/123 (9.8%) vs. 24/169 (14.2%)	AL/B1 fracture: Posterior vs. lateral	0.60	0.29–1.26	0.179
Firth logistic	9/103 (8.7%) vs. 27/189 (14.3%)	AL/B1 fracture: tapered-wedge vs. fit-and-fill	0.65	0.30–1.41	0.273
IPTW-weighted	11.43% vs. 23.99%	Any intraoperative fracture: Posterior vs. lateral	0.41	0.21–0.79	0.008
IPTW-weighted	1.95% vs. 9.42%	Greater trochanter fracture: Posterior vs. lateral	0.19	0.05–0.76	0.019
IPTW-weighted	9.48% vs. 14.57%	AL/B1 fracture: Posterior vs. lateral	0.61	0.29–1.29	0.197

Note. OR, odds ratio; CI, confidence interval; IPTW, inverse probability of treatment weighting. Conventional pooled logistic models were adjusted for surgical approach or stem geometry (as applicable), age, sex, aCCI, and CFI. The varus sensitivity model additionally included binary varus malposition (≥3°). Firth bias-reduced logistic regression was used for low-event outcomes. The IPTW propensity model estimated the probability of posterior approach from age, sex, aCCI, ASA ≥3, stem geometry, and CFI; stabilized ATE weights were truncated at the 1st and 99th percentiles. Adequate post-weighting balance was defined as |SMD| < 0.10. IPTW outcome estimates were obtained from weighted binomial generalized linear models using HC0 robust sandwich covariance.

## Data Availability

The data that support the findings of this study are available from the corresponding author upon reasonable request, subject to applicable ethical and institutional restrictions.

## Supplementary Materials

The following supporting information can be downloaded at: https://www.mdpi.com/article/10.3390/jcm15197666/s1. The completed STROBE checklist for this retrospective cohort study is provided in the Supplementary Material.

## Abbreviations

aCCI, age-adjusted Charlson Comorbidity Index; AG, Vancouver type AG greater trochanteric fracture; AL, Vancouver type AL lesser-trochanter/calcar-adjacent fracture; ASA, American Society of Anesthesiologists Physical Status Classification; ATE, average treatment effect; CBR, canal–bone ratio; CFI, canal flare index; CFRdist, distal canal fill ratio; CFRprox, proximal canal fill ratio; CI, confidence interval; CTI, cortical thickness index; CCR, canal–calcar ratio; ICC, intraclass correlation coefficient; IPTW, inverse probability of treatment weighting; OR, odds ratio; PFN, proximal femoral nail; SMD, standardized mean difference.
