# Supplementary material for: Association of Surgical Approach with Intraoperative Femoral Fracture Patterns During Cementless Hemiarthroplasty for Femoral Neck Fracture
Source: J Clin Med. 2026 Oct 3;15(19):7666. doi: 10.3390/jcm15197666 (PMC13666352; doi:10.3390/jcm15197666)
Supplement: Supplementary file 1 [file jcm-15-07666-s001.zip › jcm-4588974-supplementary.pdf]

STROBE Statement — Completed Checklist for Cohort Studies

Article: Association of Surgical Approach with Intraoperative Femoral Fracture Patterns During Cementless Hemiarthroplasty for Femoral Neck Fracture

Study design: Single-center retrospective cohort study. **Checklist basis:** STROBE cohort-study checklist. Page numbers refer to the current 16-page JCM proof.

Item	No.	STROBE recommendation (concise)	Reported on page / location
**Title and abstract**	1(a)	Identify the observational study design in the title or abstract.	p. 1 (Abstract: retrospective review); p. 3 (explicitly described as a retrospective cohort study)
**Title and abstract**	1(b)	Give an informative, balanced abstract summarizing methods and principal findings.	p. 1
**Background / rationale**	2	Explain the scientific background and rationale.	pp. 2–3
**Objectives**	3	State the specific objectives and any prespecified hypotheses.	p. 3
**Study design**	4	Present the key elements of the study design early in the Methods.	p. 3, Section 2.1
**Setting**	5	Describe the setting, location, and relevant study dates.	p. 3, Section 2.1
**Participants**	6(a)	Describe eligibility criteria and the source/method of participant selection; describe follow-up when applicable.	pp. 3–4, Sections 2.1–2.2; longitudinal follow-up was not used for the primary intraoperative outcome
**Participants**	6(b)	For matched studies, report matching criteria and numbers in each matched group.	Not applicable — this was not a matched cohort study
**Variables**	7	Define outcomes, exposures, predictors/confounders, effect modifiers, and relevant diagnostic/classification criteria.	pp. 3–7, Sections 2.1, 2.4–2.8
**Data sources / measurement**	8	State data sources and measurement/assessment methods for variables of interest; describe comparability across groups where relevant.	pp. 3–6, Sections 2.1, 2.4–2.7
**Bias**	9	Describe efforts to address potential sources of bias.	pp. 4, 7 and 13; Sections 2.2–2.3, 2.8 and Limitations
**Study size**	10	Explain how the study size was determined.	p. 6, Section 2.8 (all eligible patients in the predefined period; no a priori sample-size calculation)
**Quantitative variables**	11	Explain how quantitative variables were handled, including any categorization and its rationale.	pp. 6–7, Section 2.8
**Statistical methods**	12(a)	Describe statistical methods, including methods used to control for confounding.	pp. 6–7, Section 2.8
**Statistical methods**	12(b)	Describe subgroup and interaction analyses.	p. 7, Section 2.8
**Statistical methods**	12(c)	Explain how missing data were handled.	pp. 3–4 (complete analytical data; no additional exclusions for missing analytical data)
**Statistical methods**	12(d)	Explain how loss to follow-up was handled, if applicable.	Not applicable — the primary outcomes were intraoperative events, not longitudinal follow-up outcomes
**Statistical methods**	12(e)	Describe sensitivity analyses.	p. 7, Section 2.8 (varus-malposition sensitivity model and IPTW robustness analysis)
**Participants**	13(a)	Report numbers of individuals at each stage of the study.	pp. 3–4, Section 2.1 and Figure 1
**Participants**	13(b)	Give reasons for non-participation/exclusion at each stage.	pp. 3–4, Sections 2.1–2.2 and Figure 1
**Participants**	13(c)	Consider a flow diagram.	p. 4, Figure 1
**Descriptive data**	14(a)	Report participant characteristics and information on exposures and potential confounders.	p. 10, Table 1
**Descriptive data**	14(b)	Report the number of participants with missing data for variables of interest.	pp. 3–4 (complete baseline analytical data; no additional exclusions for missing analytical data)
**Descriptive data**	14(c)	Summarize follow-up time, if applicable.	Not applicable — the analyzed outcome was intraoperative fracture
**Outcome data**	15	Report outcome-event numbers or appropriate summary measures.	p. 8 and p. 10, Results and Table 1
**Main results**	16(a)	Report unadjusted and adjusted estimates with precision, and state the adjustment variables/rationale.	pp. 7–8 and pp. 10–11, Section 2.8, Results and Table 2
**Main results**	16(b)	Report category boundaries when continuous variables are categorized.	pp. 3, 5–7 and p. 10 (e.g., CTI < 0.40, CFI < 3.0, varus ≥ 3°)
**Main results**	16(c)	Translate relative estimates into absolute risk over a meaningful time period when relevant.	Not applicable — the study assessed intraoperative events rather than time-to-event risk
**Other analyses**	17	Report other analyses such as subgroup, interaction, and sensitivity analyses.	pp. 7–8 and pp. 10–11
**Key results**	18	Summarize the key results with reference to the study objectives.	p. 11, Discussion
**Limitations**	19	Discuss study limitations, including potential bias and imprecision.	p. 13, Discussion
**Interpretation**	20	Provide a cautious interpretation considering objectives, limitations, multiplicity, and relevant evidence.	pp. 11–14, Discussion and Conclusions
**Generalisability**	21	Discuss the generalisability / external validity of the findings.	pp. 13–14
**Funding**	22	State the funding source and the role of funders.	p. 14 (no external funding)

Note: Items concerning longitudinal follow-up are marked not applicable because the primary outcomes were intraoperative femoral fracture events. This checklist is intended as the reporting supplement for manuscript jcm-4588974.
